# Laparoscopic transabdominal-sacrococcygeal approach for resection of Altman type III sacrococcygeal teratoma in adult women: A case report

**DOI:** 10.1097/MD.0000000000037887

**Published:** 2024-04-26

**Authors:** Qiang Zhong, Qizhu Zhang, Ziwen Xiao, Hao Zhang

**Affiliations:** a Department of Gynecology, Guizhou Hospital of The First Affiliated Hospital, Sun Yat-sen University, Guiyang, China; b Department of Obstetrics and Gynecology, The Affiliated Hospital of Guizhou Medical University, Guiyang, China; c Department of Orthopaedics, The Affiliated Hospital of Guizhou Medical University, Guiyang, China.

**Keywords:** computed tomography, magnetic resonance, median sacral artery, ovarian cyst, serum tumor markers

## Abstract

**Introduction:**

Adult sacrococcygeal teratoma (SCT) is a rare disease that is not easily detected or easily missed, and its treatment is based on surgery, including transabdominal, transsacral, or a combination of both, but there are no clear guidelines for diagnosis and treatment. We share a case of Altman type III SCT in order to provide more reference protocols for the diagnosis and treatment of adult SCT, and more importantly to increase our understanding of different types of SCT cases in adults.

**Patient concerns:**

Our patient was a 31-year-old adult woman who underwent complete surgical resection of a cystic mature teratoma of the right ovary 8 years ago and is currently 13 months postpartum without menstruation, usually with a feeling of anal bulge, with symptoms such as constipation.

**Diagnosis:**

We diagnosed SCT by vaginal ultrasonography, computed tomography and magnetic resonance imaging (MRI); benign tumors were considered in the results of serum tumor markers.

**Interventions:**

We chose the surgical approach of laparoscopic transabdominal-sacrococcygeal approach to completely remove the patient SCT and coccyx.

**Outcomes:**

The location of SCT is concealed and the clinical symptoms are not obvious. Vaginal ultrasonography, CT and MRI can not only improve the diagnostic rate of SCT, but also understand the size and mass of SCT, providing an exact basis for clinicians to select the laparoscopic transabdominal-sacrococcygeal approach.

**Conclusion:**

Our sharing increases the reports of rare cases of teratoma with the same histological findings in different organ tissues of the same patient at different times, whether this occurs incidentally requires more case reports and further basic research; in addition, the laparoscopic transabdominal-sacrococcygeal approach is a safe and effective surgical approach for the treatment of Altman type III SCT in adults; finally, this case reminds us that SCT may not affect pregnancy and pregnancy outcomes and provides a reference for the selection of interventions for SCT with pregnancy.

## 1. Introduction

Teratomas arise from embryonic pluripotent germ cells and consist of cells from one or more germ cell layers (ectoderm, mesoderm, and endoderm) and can occur anywhere in the body.^[1]^ The coccyx is a primordial germ cell-enriched site, so the sacrococcygeal region is a common site of teratoma in infants and childhood; According to the site of sacrococcygeal teratoma (SCT), Altman et al classified it into 4 categories: type I (exposed type): the tumor is completely outside; type II (mixed internal and external type): the tumor is located in front of the sacrum and grows into the pelvic cavity and buttock at the same time; type III (dumbbell-shaped internal and external mixed type): the tumor straddles around the sacrum, mainly the presacral tumor, and rectal and urethral compression symptoms often occur; type IV (occult type): the tumor is located only in the presacral region, grows only into the pelvic cavity, and does not present a mass in vitro.^[2]^However, SCT is extremely rare in adults, with an incidence of 1 in 40,000 to 1 in 63,000 and a male-to-female ratio of 1:3.^[1]^ Most adult SCTs are type III or IV, and there are currently no clear guidelines for diagnosis and treatment.

## 2. Case presentation

**Figure 1.**
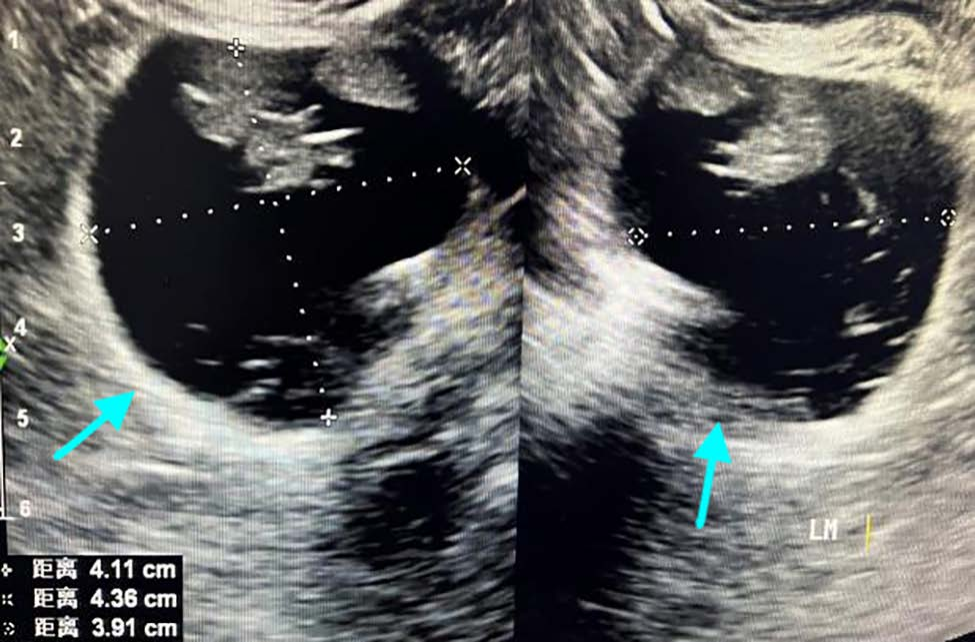
Vaginal ultrasound showed a mixed echogenic mass behind the rectum, and the echogenicity showed dough sign and short line sign without blood flow signal, considering mature teratoma.

**Figure 2.**
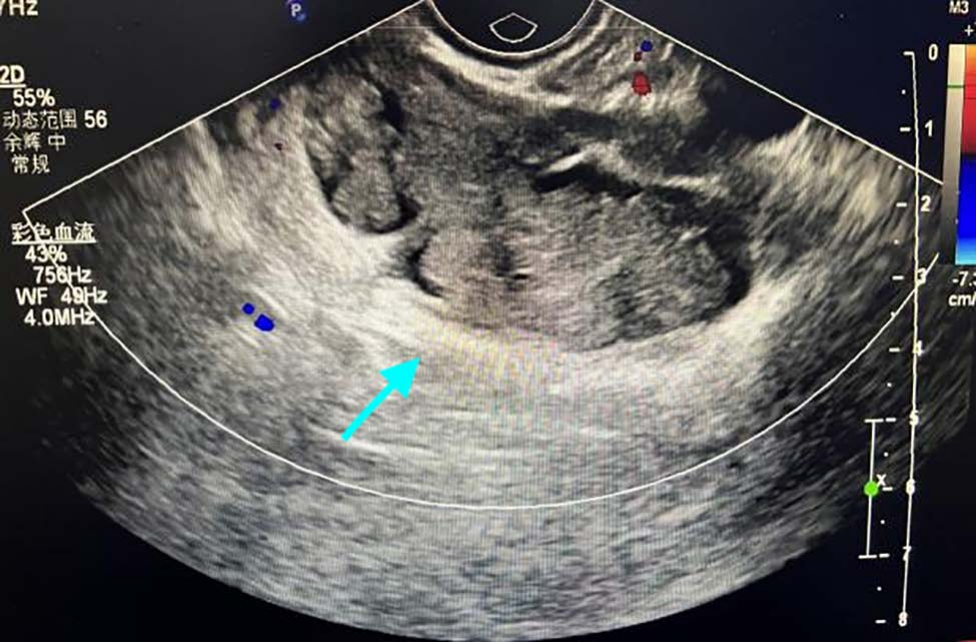
Vaginal ultrasound showed a mixed echogenic mass behind the rectum, and the echogenicity showed dough sign and short line sign without blood flow signal, considering mature teratoma.

**Figure 3.**
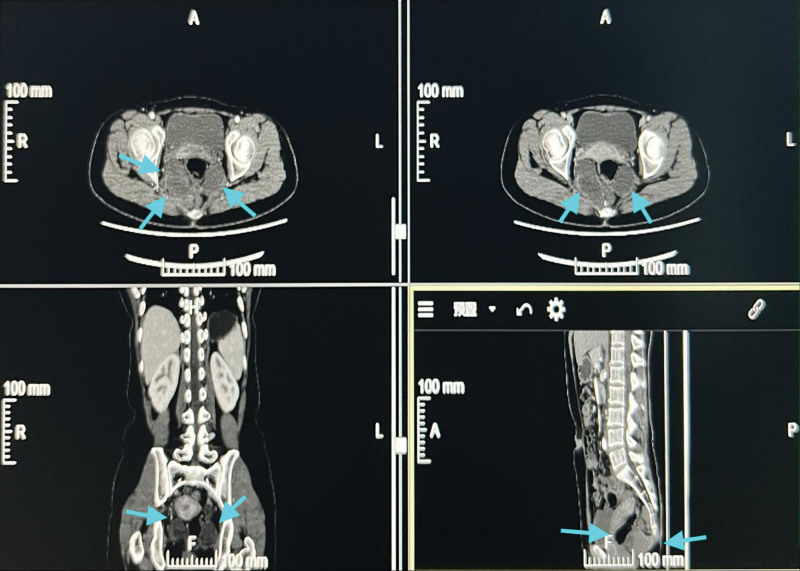
Enhanced CT showed multiple multilocular cystic increased density shadows in the sacrococcygeal region, which were irregular in shape and clear in border, calcified shadows were observed at the edge of some lesions, and the surrounding fat space was clear, which was considered to be cystic mature teratoma. CT = computed tomography.

**Figure 4.**
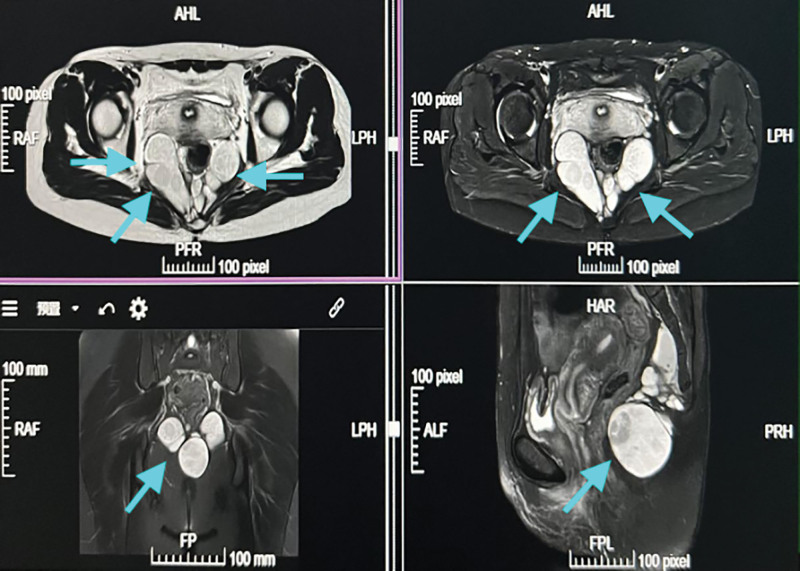
Pelvic MRI showed multiple sacrococcygeal teratomas in the sacrococcygeal region, the larger one was located 10,000 behind the rectoanal canal, fluid levels were observed in some lesions, and the rectum was compressed and moved anteriorly. MRI = magnetic resonance imaging.

The patient, 31 years old, G2P1, delivered a healthy baby girl at term in March 2022 and visited the hospital for diagnosis and treatment due to “no menstruation 13 months after delivery”; She usually had a feeling of anal bulge, with constipation, and no other symptoms. Pelvic examination: On bimanual examination, an active palpable mass in the left adnexal area measuring approximately 4 × 3 × 3 cm was noted; On rectovaginal examination, a poorly mobile soft scar measuring approximately 5 × 3 × 3 cm and 5 × 4 × 4 cm was present in the left and right anterior sacrum, respectively. Vaginal ultrasound (Figs. 1 and 2), Total Abdominal Enhanced CT (Fig. 3), and magnetic resonance imaging (MRI) plain scan (Fig. 4) revealed multiple teratomas with benign signs growing along the sacrococcygeal bone behind the rectum. Relevant serum tumor markers: alpha-fetoprotein 1.79 ng/mL, carbohydrate antigen 12-5 70.71 U/mL, and CA19-9 14.98 U/mL. Past medical history: In 2015, the patient underwent “laparoscopic bilateral ovarian cystectomy” due to “bilateral ovarian cysts.” Histopathological examination showed (left ovarian cyst wall) simple cyst; (right ovarian cyst wall) cystic mature teratoma.

**Figure 5.**
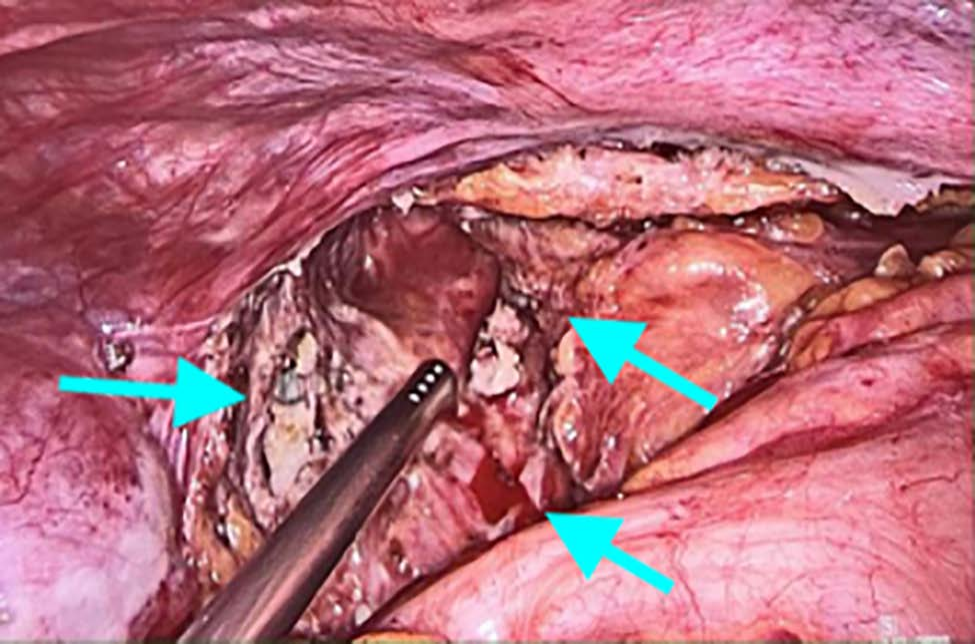
SCT located in the pelvic part as seen in laparoscopic surgery. SCT = sacrococcygeal teratoma.

**Figure 6.**
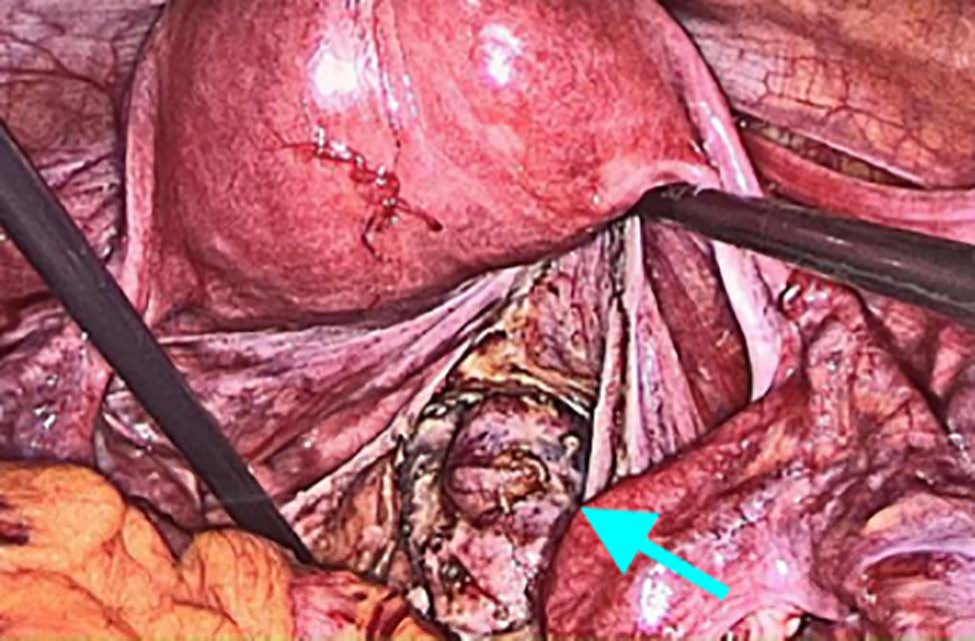
SCT located in the pelvic part as seen in laparoscopic surgery. SCT = sacrococcygeal teratoma.

**Figure 7.**
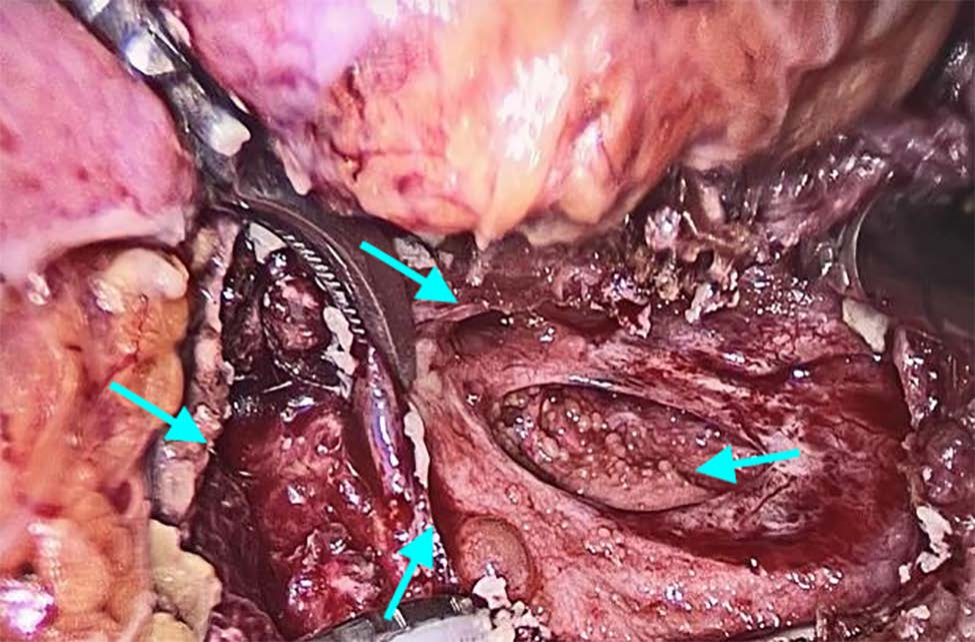
SCT located in the pelvic part as seen in laparoscopic surgery. SCT = sacrococcygeal teratoma.

**Figure 8.**
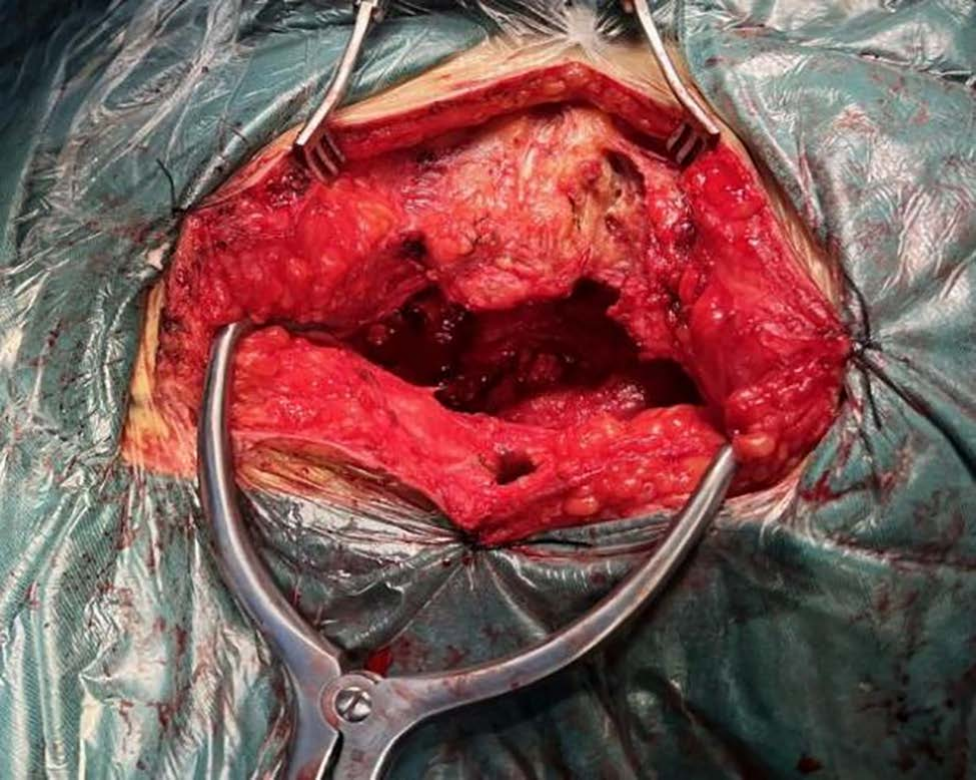
Inverted Y-shaped longitudinal incision via para sacrococcygeal approach.

**Figure 9.**
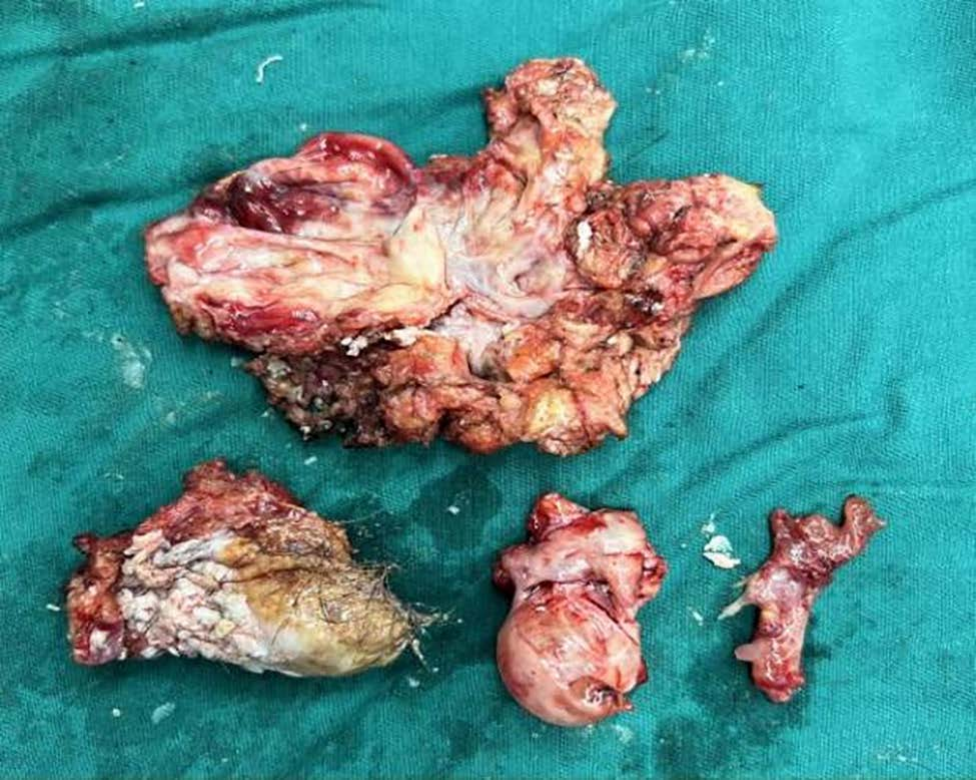
Surgically resected SCT specimen. SCT = sacrococcygeal teratoma.

**Figure 10.**
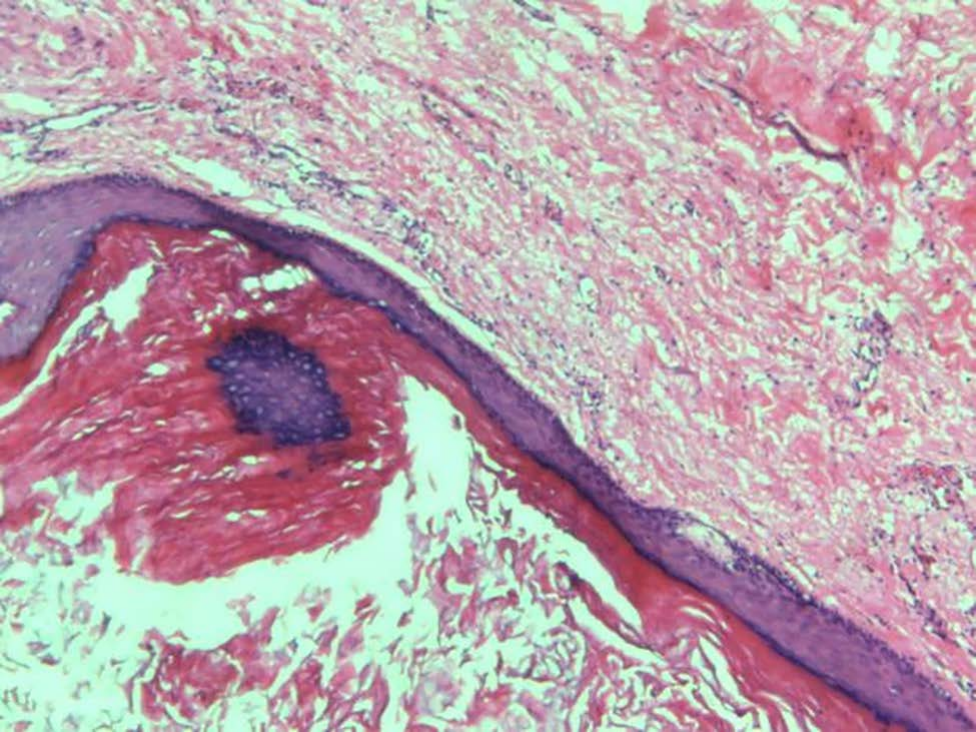
The surface of fibrous tissue was covered with stratified squamous epithelium and keratoses.

**Figure 11.**
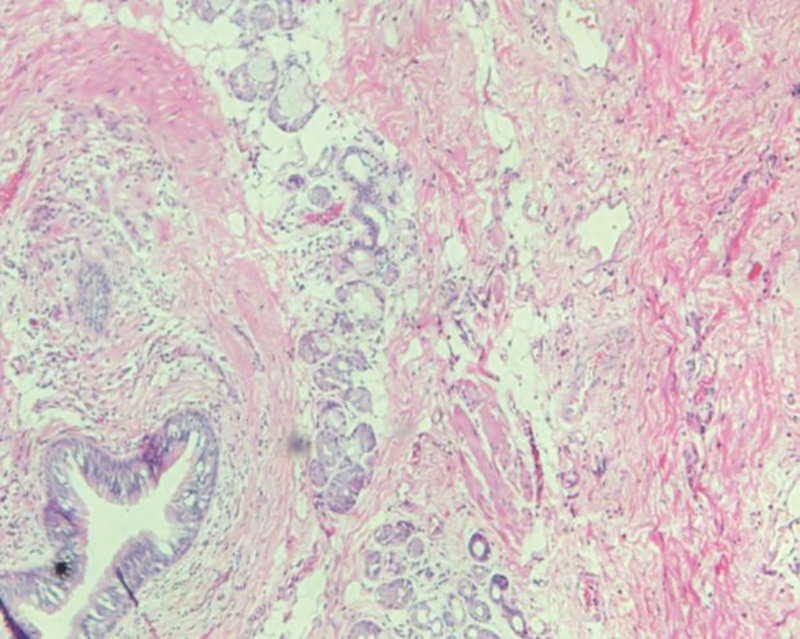
Nested glands were observed in fibro adipose tissue, and glandular tube-like structures composed of columnar epithelium were observed.

Combined with the patient medical history, physical examination and auxiliary examination, the patient was diagnosed with SCT, benign and malignant to be excluded. The patient had a large SCT volume, irregular multilocular shape, wide range of invasion into the pelvic cavity, reaching the deep posterior peritoneum, and compressing the rectum. In order to completely remove the tumor and avoid recurrence, we selected a laparoscopic combined transabdominal-sacrococcygeal approach for surgical planning. We first performed laparoscopic exploration to understand teratoma morphology, location, and relationship with various organs in the pelvic cavity. Intraoperative findings: Multiple cystic soft tumors protruding into the pelvic cavity were detected in front of the left sacroiliac joint, left and right sides of the sacrococcygeal bone and posterior to the anterior sacrum rectum, respectively, and pushed along the third sacrum to the fifth sacral vertebral body to grow, rooted in the deep posterior peritoneum, with poor mobility, and the size of the mass protruding into the pelvic cavity was about 1 × 1 × 2 cm, 4 × 3 × 2 cm, 5 × 3 × 3 cm, and 4 × 3 × 2 cm, respectively (Figs. 5–7). Subsequently, along the paracolic gutter, laparoscopically, from the left and right sides of the sacrum from top to bottom, until the levator ani muscle, opened the posterior peritoneum, and found that the mass was irregular in shape, closely adhered, and had no obvious space with the surrounding tissue fascia, separated the convex pelvic part of the tumor, and exposed the anatomical location of the bilateral ureters and median sacral artery. During the operation, the tumor fold was punctured, milky fat-like fluid secretion flowed out, a small amount of hair-like tissue was observed, multiple cysts were observed, and cauliflower-like vegetations were observed in some cysts (Figs. 5 and 7). Subsequently, the patient position was changed and an inverted “Y” longitudinal incision was made from S1 to the posterior midline of the coccyx (Fig. 8) to expose the spinous process and coccyx of the S3 to S5 laminae. A cystic tumor measuring about 3 × 2 × 2 cm was observed behind the S5 lamina, the S5 lamina was not destroyed, and the tumor and coccyx were completely removed. Expose the anterior sacrum, distal coccyx and posterior rectum, a cystic and solid tumor was observed in the anterior left sacroiliac joint, left and right side of retroperitoneal sacrum, respectively, which were multilocular and about 3 × 3 × 4 cm, 7 × 5 × 12 cm and 13 × 11 × 6 cm in size; the latter 2 were externally located at the inner edge of S4 nerve root, the upper boundary was located at the upper edge of S5 vertebral body, and the lower boundary was located at the upper edge of levator ani muscle. A cystic and solid tumor measuring about 8 × 9 × 10cm was observed behind the anterior sacrum rectum, with the upper boundary located at the upper edge of the coccyx, the lower boundary located at the upper edge of the levator ani muscle, and the posterior boundary located in the deep fascial layer below the coccyx. We performed complete resection of the above tumors that were completely exposed after laparoscopic dissection via the para-coccygeal approach (Fig. 9). The total intraoperative blood loss was 400 mL, and the patient was discharged uneventfully 10 days after the end of surgery without any complications. Histopathology of the tumor specimen after surgery showed: (SCT) consistent with cystic mature teratoma; (pelvic tumor cyst wall) consistent with cystic mature teratoma (Figs. 10 and 11).

Follow-up: 3 months after the end of surgical treatment, the patient came back to the hospital for reexamination, blood HCG showed pregnancy status, vaginal B ultrasound showed intrauterine pregnancy, live fetus; no SCT imaging findings were observed.

## 3. Discussion

Adult female patients with SCT are generally asymptomatic, and some patients are often missed, but it is a slow-growing cystic tumor that is often detected by CT and MRI when it reaches a certain size and causes compression symptoms in the pelvic cavity and abdominal cavity. Our patient confirmed the occult nature of adult SCT, and although preoperative imaging showed multiple large tumors in the sacrococcygeal region, the largest of which was about 13 × 11 × 6 cm in size on intraoperative exploration, she did not present with significant symptoms of compression of surrounding organs; in addition, she did not present with symptoms of neurological dysfunction such as numbness, pain, or paresthesia of the lower extremities. The actual prevalence of SCT in adults may be more than has been reported, and how to reduce the increased rate of malignant transformation due to prolonged history due to missed diagnosis is a topic of concern.

Because of the complex and cryptic anatomy of the sacrococcygeal region, the diagnosis of adult SCT depends on the patient clinical presentation, physical examination, and imaging studies. However, cystic mature teratomas grow slowly, with an average growth rate of 1.8 mm/year in premenopausal women,^[1]^ and most patients are unable to identify the size and location of the tumor by physical examination; We usually need color Doppler ultrasonography, CT and MRI to help us evaluate the size, extension direction of pelvic tumors and their anatomical relationship with surrounding tissues and organs, and then preliminarily determine the benign and malignant tumors by serum tumor markers: alpha-fetoprotein, CA-199, HE4, and CA-125. However, these tests still have their limitations. According to our patient medical history and all auxiliary examinations, we believe that these multiple SCTs have been present for at least 5 years or more. What puzzles us is that this is not the case. Our patient was pregnant 15 months before the diagnosis of SCT, and neither prenatal examination during pregnancy nor obstetric examination before delivery revealed data supporting this diagnosis nor physical examination descriptions, and although she usually had rectal compression symptoms, it had not affected her normal life and the progress of vaginal delivery in term pregnancy. This is an unusual case report and we suggest 2 possible conjectures: During pregnancy, the tumor is occluded by the intrauterine fetus or missed due to factors such as limited detection means and technical level; After the end of pregnancy, SCT rapidly increases in size in a short period of time under the influence of certain factors, resulting in an increase in tumor size. Because of objective factors, we could not determine whether there was a link between pregnancy and SCT. Fortunately, our patient did not have complications such as rupture and bleeding due to fetal compression of the tumor during vaginal delivery, nor dystocia due to abnormal birth canal due to SCT. The above experience tells us that CT and MRI of the corresponding site should be performed promptly in patients with suspected SCT when conditions permit.

Cystic mature teratoma is a benign tumor with a good prognosis, but there is also a possibility of recurrence, and there is a malignant transformation rate of 1% to 2%, which increases with the age of recurrence. Padilla reported a recurrence rate of 13% in SCT, 60% of which occurred after 3 years after primary surgical resection, and even half of all malignant recurrences came from what we considered benign mature teratomas.^[3]^ In addition, there are also large differences in the time and location of SCT recurrence. Okino reported a 45-year-old woman who underwent oophorectomy on the affected side 34 years ago for a mature cystic teratoma of the left ovary, which was found in the right diaphragm only 1 year later.^[4]^ Recently, Velayos also reported a sacrococcygeal tumor resection in the neonatal period, which developed the tumor again on the ovary 12 years later, and finally found that the histopathology of the tumor specimen showed mature cystic teratoma after both operations.^[5]^ Our patient had undergone complete surgical stripping 8 years ago for cystic mature teratoma of the left ovary and simple cyst of the right ovary, and no tumor recurrence was found at the same site during this laparoscopic exploration. These position-specific teratomas are formed at specific times and conditions by germ cells that fail to migrate to normal positions, and whether there is an association between them requires extensive basic research and more similar case reports, and we believe that they are more like independent events. The treatment of choice for SCT is complete surgical resection, and the main surgical approach usually includes transabdominal, transsacral, or a combination of both, although there are no clear selection criteria, the size and location of the tumor are important reference indications for the surgical approach. Alyousef reported a 16-year-old patient with Altman type IV sacrococcygeal cystic mature teratoma, approximately 11 cm in diameter, who underwent complete resection of the tumor by laparoscopy combined with transsacral surgery.^[6]^ Osei also reported the successful completion of median sacral artery dissection and tumor resection by laparoscopic combined posterior sagittal approach in 2 children with Altman type III and IV SCT.^[7]^ Laparoscopy can create the best surgical field, accurately identify and ligate the vascular structure of the primary tumor, free the boundary of the pelvic tumor, and confirm the depth of the tumor; the sacrococcygeal approach can completely remove the tumor. Therefore, we chose the laparoscopic combined transabdominal-sacrococcygeal approach, and the final surgery also reached our expected results, avoiding the occurrence of complications such as nerve injury, massive hemorrhage and infection.

Currently, there remains debate as to whether the coccyx should be removed at the time of SCT surgery. The coccyx has full potential cells, and the risk of recurrence as well as the rate of malignant transformation associated with SCT may be associated with the absence of coccygectomy for surgery. Cui showed that the risk of recurrence can be as high as 30% to 40% if the SCT tumor is removed without removing the coccyx^[8]^; Vana have reported a case of SCT that was surgically removed in the neonatal period, which recurred 31 years later and showed adenocarcinoma on postoperative histopathology, and they concluded that preservation of the coccyx at the first operation may be the cause of recurrence and malignant transformation.^[9]^ These case reports remind us that thorough surgery, including complete resection of the SCT and coccyx, is an important means of preventing SCT recurrence.

There are few published reports on SCT in adult women, and our case sharing provides new differences, while the laparoscopic transabdominal-sacrococcygeal approach is a safe and classic surgical approach for adult Altman type III SCT; However, our report also has limitations, the patient lacks regular physical examination reports and prenatal examination data during the last pregnancy are missing, and we cannot accurately determine whether she suffers from SCT during pregnancy or even earlier. Through literature review, according to the growth rate of teratoma and the patient condition description, we infer that the patient suffers from SCT this time is not after the end of the last pregnancy, which reminds us that for patients with a previous history of teratoma, long-term regular follow-up should be adhered to after surgical resection, and imaging examinations such as ultrasound, CT, and MRI are important follow-up and screening methods, and are also important basis for the surgical team to develop the extent of resection and surgical methods before surgery. Finally, our reported patient presented with an intrauterine pregnancy 3 months after surgery and we will follow-up closely and we need more reports of SCT in specific case types.

## Author contributions

**Formal analysis:** Qiang Zhong, Qizhu Zhang.

**Investigation:** Qiang Zhong.

**Methodology:** Hao Zhang, Ziwen Xiao.

**Project administration:** Qiang Zhong, Ziwen Xiao.

**Resources:** Qiang Zhong, Hao Zhang, Ziwen Xiao.

**Writing – original draft:** Qiang Zhong.

**Writing – review & editing:** Qiang Zhong, Ziwen Xiao, Qizhu Zhang.
